# TiO_2_ Nanoribbons/Carbon Nanotubes Composite with Enhanced Photocatalytic Activity; Fabrication, Characterization, and Application

**DOI:** 10.1038/s41598-018-19172-w

**Published:** 2018-01-15

**Authors:** Mohamed Shaban, Abdallah M. Ashraf, Mostafa R. Abukhadra

**Affiliations:** 1 Nanophotonics and Applications Lab, Physics Department, Faculty of Science, Beni-Suef University, Beni-Suef, 62514 Egypt; 2 Chemistry Department, Faculty of Science, Beni-Suef University, Beni-Suef, 62514 Egypt; 3 Geology Department, Faculty of Science, Beni-Suef University, Beni-Suef, 62514 Egypt

## Abstract

TiO_2_ nanoribbons (TiO_2_ NRs) loaded with FeCo-Al_2_O_3_ catalyst were synthesized and used as a precursor in the synthesis of TiO_2_ nanoribbons/carbon nanotubes (TiO_2_ NRs/CNTs) composite by a chemical vapor deposition (CVD) method. TiO_2_ NRs and TiO_2_ NRs/CNTs composite were characterized by XRD, FT-IR, TEM, SEM, EDX and UV-Vis spectrophotometer. The results revealed the formation of TiO_2_-B and hydrogen titanate nanoribbon like structures by the hydrothermal treatment. After loading TiO_2_ NRs by FeCo-Al_2_O_3_ catalyst and the CVD growth of carbon nanotubes, the synthetic TiO_2_ nanoribbons converted entirely to TiO_2_-B nanoribbons with nanopits structure. The composite composed of tube-like nanostructures forming an interlocked network from CNTs and TiO_2_-B NRs. The composite shows a relatively red-shifted band gap (3.09 eV), broader and stronger UV absorption band relative to TiO_2_ NRs. The photocatalytic properties of TiO_2_ NRs and TiO_2_ NRs/CNTs composite were studied under sunlight irradiation. The photocatalytic degradation of methylene blue (MB) dye was investigated as a function of contact time, dye concentration, and catalyst dose. The kinetics and mechanisms of degradation were discussed. TiO_2_ NRs/CNTs composite showed higher stability after six runs and 50% shorter irradiation time than TiO_2_ NRs photocatalyst.

## Introduction

Approximately 100000 types of dyes are produced with an annual production rate of over 7 × 10^5^ to 1 × 10^6^ tons and used in several industries such as textile, leather, paper, printing, paint, pigments, rubber and plastic^1^. About 10 to 15% of the used dyes discharged into the surrounding environment and water bodies which cause allergy, dermatitis, cancer, skin irritation, dysfunction of kidneys, liver and reproductive system in humans^2^. Methylene blue is one of the commonly used cationic dyes that are harmful to human beings. It can cause eye irritation, skin, and respiratory tract irritation. Also, it can create permanent injury to the cornea and conjunctiva in human and rabbit eyes^3^. One of the commonly used dyes is methylene blue dye which has a wide range of medical applications including several diagnostic and therapeutic procedures^4^. It is commonly used as anti-haemoglobinemia, redox agent, antidote, antiseptic, disinfectant and stain for bacteria^4^. Also, methylene blue was used as pigments for several materials such as rubber, papers, and textiles^5^. Annual discharging of wastewater contaminated by methylene blue dye causes several environmental problems including increasing the level of chemical oxygen demand above the limit which may cause the death of the present aquatic organisms^6^.

Several techniques have been investigated to remove dyes from water and industrial wastewater including chemical precipitation, conventional coagulation, reverses osmosis, ion exchange, electrodialysis, electrolysis, adsorption, and photocatalytic degradation^7^. Among the used techniques for the removal of dyes, adsorption and photocatalytic degradation are recommended as environmentally, cheap and efficient methods^8^. However, adsorption by low-cost materials is efficient in dye removal, but such method produces a lot of solid wastes^9^.

Environmental heterogeneous photocatalytic materials were preferred in degradation of dyes. The main advantage of using heterogeneous photocatalysts is its ability to profiteer the solar energy in the production of hydroxyl radicals for dye oxidation^8^. Several inorganic materials of suitable band gap energy have been studied for photocatalytic degradation of dyes including several semiconductor metal oxides^10^. Among Such metal oxides, TiO_2_ materials of different forms (powder, Nanotubes, Nanorods, and Nanoribbons) exhibit high efficiency in photocatalytic degradation of dyes^11^. TiO_2_ photocatalysts characterized by high stability, availability, cheap, strong oxidation power, non-toxic and excellent band gap (3–3.2 eV) without modification^10^.

The photocatalytic properties of TiO_2_ materials depend mainly on their phase composition, particle size, doping, surface area, and morphology^12,13^. Several authors focused on the enhancement of the photocatalytic properties of TiO_2_ materials through several modification processes or fabrication of TiO_2_ based nanocomposites^11^. Modification of TiO_2_ was performed through different methods including doping of TiO_2_ by metals, non-metals, semiconductors nanoparticle, graphene, and carbon nanotubes (CNTs)^11,14^. In particular, carbon nanotubes (CNTs) became desirable material for its specific mechanical, physical, chemical, electroconductive, and field emission properties^15,16^. So, there is considerable interest in the synthesis of hybrid materials from TiO_2_ nanomaterials and CNTs as enhanced production of high photocatalytic efficiency^14,17^. Several methods were used in the synthesis of TiO_2_/CNTs composites involving random mixing of CNTs with TiO_2_ particles, a coating of CNTs with TiO_2_ nanoparticles, and warping of CNTs around the TiO_2_ nanoparticles^18^. Tarigh *et al*. succeeded in the synthesis of multi-walled CNTs/TiO_2_ nanoparticles composite through electrostatic attraction in ethanol solution^19^. Also, CNTs/TiO_2_ nanotubes composite was synthesized by mixing CNTs and TiO_2_ nanotubes in the nitric acid washing process. Zhang *et al*. reported that CNTs/TiO_2_ nanotubes composite could be synthesized successfully through a hydrothermal method in an autoclave followed by heating for 2 h at 150 °C^20^. The combination of them leads to transfer of a photoexcited electron from TiO_2_ to the carbon material and hinder the recombination processes which enhance the oxidative reactivity of the composite^21^. Moreover, it is reported that TiO_2_/carbon composites exhibit improvement in the performance under the visible light. This attributed to the increase in the absorption in the visible light range and the lifetime of the carrier^22^.

Besides TiO_2_ nanotubes, different other morphologies such as TiO_2_ nanoribbon, nanorods, nanowires, and nanosheets attracted attention due to a variety of their applications in photocatalysis, photovoltaic cells, lithium-ion batteries, and sensors. Especially, TiO_2_ nanoribbons structure possess high porosity and high surface area due to the formation of the mesopores and nanocavities on their surfaces^23^. Moreover, Santara *et al*. reported that the porous structure of the nanoribbons strongly improved the electrochemical properties and the ferromagnetic ordering of the negative electrode in lithium-ion batteries^24^. Tao *et al*. showed that TiO_2_ nanoribbon spheres possessed higher photocatalytic activity than TiO_2_ nanoparticles due to the decreased recombination rate of photogenerated electron-hole pairs and vast surface area^25^. Although the important properties of the TiO_2_ nanoribbons, their photocatalytic property still need more improvement. To the best of our knowledge, there is no report on the fabrication, characterization, optical, and photocatalytic properties of TiO_2_ nanoribbons/CNTs nanocomposites.

For the first time, in this work, we investigate the synthesis of TiO_2_ nanoribbons (NRs)/Carbon nanotubes (CNTs) nanocomposite through a novel method by loading of FeCo-Al_2_O_3_ CNTs growth catalyst on TiO_2_ NRs followed by chemical vapor deposition of CNTs. The structures, morphologies, and optical properties of the fabricated nanostructures were investigated. The photocatalytic activity of the raw TiO_2_ NRs and the synthetic composite in the degradation of methylene blue (MB) dye was examined under sunlight in a comparative study. The photocatalytic degradation was investigated as a function of contact time, dye concentration, and catalyst dose. Also, degradation kinetics and mechanisms were addressed. Moreover, the catalyst stability was investigated. Sunlight as a natural source of light was selected in this study for two reasons; the first is related to the economic value of using natural sunlight instead of artificial sources and the second reason is related to testing the applicability of the product to be used in natural surface water bodies without any special stations.

## Results and Discussion

### Structural properties

**Figure 1:**
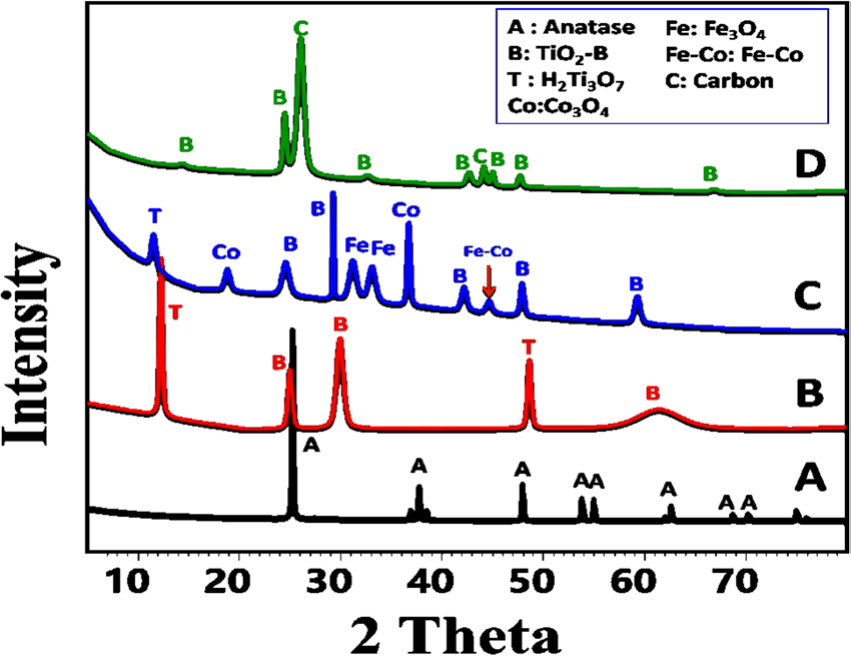
XRD patterns of (**A**) raw TiO_2_ powder, (**B**) hydrothermally synthesized TiO_2_ nanoribbons, (**C**) TiO_2_ nanoribbon supported by the FeCo-Al_2_O_3_ catalyst, and (**D**) TiO_2_-B NRs/CNTs nanocomposite.

XRD patterns for the starting inactivated TiO_2_ powder and the synthetic TiO_2_ based nanostructures are shown in Fig. 1. The starting TiO_2_ powder, Fig. 1A, composed of anatase as the dominant phase. The main characteristic peaks of tetragonal TiO_2_ anatase at 2theta 25.3°, 37.8°_,_ and 48° are related to (101), (004) and (200) crystal planes, respectively (JCPDS card No. 21–1272)^26^. In addition, other related peaks appear at 48.1°(200), 53.8° (105), 55 ° (211) and 62.6 ° (204). The hydrothermal treatment of TiO_2_ powder with sodium hydroxide solution for 24 h at 170 °C followed by partial calcination at 450 °C for 2 h resulted in the formation of mixed phases from hydrogen titanate and TiO_2_-B nanoribbons with monoclinic crystal system (Fig. 1B). The hydrothermal treatment process resulted in the formation of sodium titanate nanoribbons. Sodium titanate was converted into layered hydrogen titanate during the washing process by HCl acid (0.1 N). The present Na^+^ ions in sodium titanate (Na_2_Ti_3_O_7_) was replaced by H^+^ ions in HCl and resulted in the formation of hydrogen titanate (H_2_Ti_3_O_7_)^27^. Under the effect of partial calcination, the present hydrogen titanate was transformed partially into TiO_2_-B nanoribbons. Peaks at 11.43° and 48.67° are ascribed to the formation of hydrogen titanate and assigned to (200) and (020) crystalline planes of monoclinic crystals, respectively (PCPDF card No. 44–0131) (Fig. 1B). Additionally, the characteristic peaks of TiO_2_-B nanoribbon appeared at 24.9° and 29.8° and attributed to (110) and (−401) planes, respectively (JCPDS card No. 74–1940) (Fig. 1B).

The XRD pattern of the FeCo-Al_2_O_3_ catalyst supported on TiO_2_ NRs appears in Fig. 1C. The resulted pattern reflected the presence of TiO_2_-B as the dominant TiO_2_ phase at 2theta 24.85° (110), 29.72° (−401), 33.18° (310), 41.7° (−203), 47.8° (−512) and 59.2° (004). The other peaks are related to the precipitated catalyst which composed of Co_3_O_4_ (JCPDS card No. 01-071-0816), Fe_3_O_4_ (JCPDS, No. 00-003-0863) and Fe-Co as indicated in XRD charts in Fig. 1.

Hydrogen titanate also was detected as a single peak at 2theta 11.27° (200) (PCPDF card No. 44-0131) (Fig. 1C). The reduction in the number of hydrogen titanate peaks may be attributed to its transformation to TiO_2_-B with the calcination of the catalyst supported TiO_2_ nanoribbons at 450 °C for 4 h. The recorded shifting in the position of the characteristic peaks of TiO_2_-B to lower angles is related to the compressive stress of the precipitated iron and cobalt oxides and the effect of calcination process.

The XRD pattern of the final synthesized TiO_2_ NRs/CNTs nanocomposite appears in Fig. 1D. The results reflected the complete conversion of hydrogen titanate nanoribbons into TiO_2_-B nanoribbons during the formation of CNTs under temperature 700 °C for 50 min. The characteristic peaks of TiO_2_-B nanoribbons were detected at about 14.23°, 24.48°, 32.66°, 42.66°, 44.12°, and 47.69° (JCPDS card No. 74-1940)^23^. Whereas, the characteristic diffraction peaks of carbon nanotubes were detected at 26.05° (002) and 44.12° (100), which are in agreement with previously reported results^28^. The characteristic peaks of TiO_2_-B and CNTs in the composite show noticeable shifts from the original positions of the individual components reflecting the interaction between them in the formed composite.

The structural parameters of un-activated TiO_2_ powder, the synthetic TiO_2_ NRs, catalyst/TiO_2_ NRs, and TiO_2_ NRs/CNTs nanocomposite were illustrated in Table 1. The average crystallite size (D) was estimated according to the Scherrer equation (D = (0.9λ)/W Cos (θ)). Where W is in radians, θ is the Bragg’s angle, and λ is the X-ray wavelength (CuKα = 0.15405 nm). As displayed in Table 1, there is a noticeable decrease in the average crystallite size with the conversion of TiO_2_ anatase to hydrogen titanate nanoribbons and TiO_2_-B nanoribbons. Hydrogen titanate shows no apparent changes in the crystallite size after the coprecipitation of FeCo-Al_2_O_3_. TiO_2_-B nanoribbons exhibit a definite increase in the crystallite size from 10.26 to 15.39 nm and 19.616 nm with the coprecipitation of the catalyst and formation of the TiO_2_ NRs/CNTs nanocomposite.

**Table 1:** XRD parameters of the starting TiO_2_ powder, the synthetic TiO_2_ NRs, catalyst/TiO_2_ NRs, and TiO_2_ NRs/CNTs nanocomposite.

Compound		Peaks		D (nm)	δ(dislocation/nm^2^)	Texture Coefficient	Micro Strain%
		2θ	Planes				
TiO_2_ Raw		25.3°	(101)	44.26	5.1047 × 10^−4^	2.105	0.267380
		37.8°	(004)	45.15	4.9 × 10^−4^	0.3859	0.210517
		48°	(200)	40.50	6.09 × 10^−4^	0.5091	0.082071
TiO_2_ Nanoribbons	H_2_Ti_3_O_7_	11.43°	(200)	17.72	3.184 × 10^−3^	1.3731	1.233250
		48.67°	(020)	17.032	3.448 × 10^−3^	0.6268	0.370480
	TiO_2_ – B	24.9°	(110)	14.86	4.528 × 10^−3^	1.057	0.908951
		29.8°	(−401)	10.11	9.783 × 10^−3^	1.601	1.264481
		61.8 °	(−802)	1.972	25.71 × 10^−2^	0.341	3.656473
TiO_2_ Nanoribbons/Catalyst	H_2_Ti_3_O_7_	11.27°	(200)	16.55	3.65 × 10^−3^	—	2.162353
	TiO_2_ – B	24.85°	(110)	10.127	9.764 × 10^−3^	0.6657	1.612603
		29.72°	(−401)	55.10	3.29 × 10^−4^	2.87	0.131953
		33.18°	(310)	9.677	10.67 × 10^−3^	0.71799	1.182843
		41.7°	(−203)	13.82	5.235 × 10^−5^	0.487	0.681909
		47.8 °	(−512)	19.75	2.56 × 10^−3^	0.687	0.391862
		59.2°	(004)	14.17	4.980 × 10^−3^	0.5701	0.462148
TiO_2_ NR/CNTs	CNTs	26.05°	(002)	10.680	8.7671 × 10^−3^	1.746	1.423243
		44.12°	(100)	21.37	2.189 × 10^−3^	0.253	0.429468
	TiO_2_ – B	14.37 °	(001)	3.88	66.42 × 10^−3^	0.188	2.785129
		24.48 °	(110)	21.613	2.14 × 10^−3^	3.76	0.699158
		32.66 °	(310)	10.02	9.96 × 10^−3^	0.294	1.199121
		42.66 °	(003)	14.39	4.829 × 10^−3^	0.8306	0.673236
		44.98 °	(−403)	23.53	1.80 × 10^−3^	0.974	0.312200
		47.69 °	(−512)	19.992	2.5 × 10^−3^	0.769	0.393770
		66.82 °	(711)	11.53	7.52 × 10^−3^	0.1821	0.465374

Also, the average microstrain was estimated and listed in Table 1. The positive sign of microstrain indicates that the stress in the starting powder, the synthetic nanoribbons, and carbon nanotubes are tensile in nature. For more information about the number of defects, starting powder, the nanoribbons, catalyst/TiO_2_ NRs, and TiO_2_ NRs/CNTs nanocomposite, the dislocations density (${\rm{\delta }}$) is estimated using Williamson and Smallman’s relation, $\delta =\frac{N}{{D}^{2}}$^29^. Where N is a factor equals unity for the minimum dislocation density^30^. The values of minimum $\delta$ are listed in Table 1. The dislocations density for the TiO_2_ – B is higher than that of H_2_Ti_3_O_7_ phase. This may be related to the transfer of the layered H_2_Ti_3_O_7_ nanoribbon to TiO_2_ – B nanoribbon and the formation of the nanopits in its surface^31^. This refers to the partial calcination of H_2_Ti_3_O_7_ to TiO_2_ – B_._

The preferred orientations of the composite are evaluated by the texture coefficient (TC) of the *hkl* planes. TC(*hkl*) is calculated from the X-ray data using the well-known formula

1 $${TC}({hkl})=\,\frac{{{I}}_{{r}}({hkl})}{\frac{1}{{N}}{\sum }^{}{{I}}_{{r}}({hkl})}$$

where *I*_*r*_ = $\frac{I(hkl)}{{I}_{o}(hkl)}\,\,$ is the ratio between the measured intensity, I(*hkl*), and the corresponding standard intensity *I*_*o*_ *(hkl) for the plane hkl*, *N* is the number of reflections. TC of the composite and its individual constitutes were calculated and the obtained values are listed in Table 1. It is observed from Fig. 1 and Table 1 that the raw TiO_2_ show preferred orientation along (101) direction. The hydrothermally formed TiO_2_ nanoribbons show a prefered orientation along (200) for H_2_Ti_3_O_7_ and two prefered orintions along (110) and (−401)for TiO_2_ – B. After the formation of the catalyst, the only prefered orination was (−401) for TiO_2_- B. Whereas (110) plane becomes the prefered orintation for TiO_2_- B and (002) for CNTs in the final TiO_2_ NRs/CNTs composite.

### Morphological and Textural characterization

**Figure 2:**
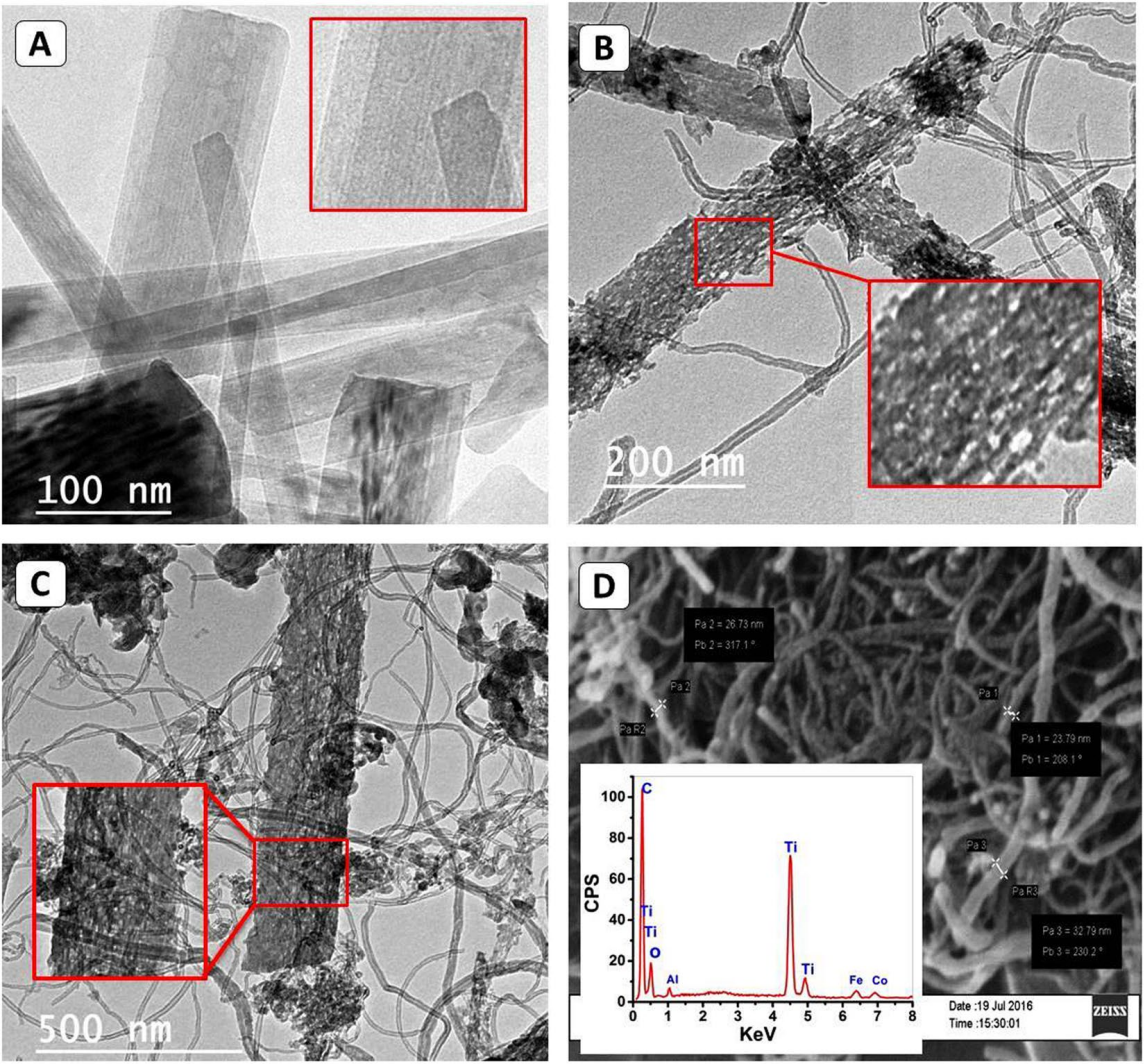
TEM images of hydrothermal synthetic TiO_2_ NRs (**A**), the formed nanopits throughout the surface of ribbon (**B**), an intergrowth of CNTs with TiO_2_-B nanoribbons (**C**), and SEM image and inset EDX spectrum of TiO_2_ NRs/CNTs composite (**D**).

Surface morphology and the internal structure of the synthetic TiO_2_ nanoribbons and TiO_2_(NRs)/CNTs nanocomposite were characterized by FE-SEM and HR-TEM images as shown in Fig. 2. HR- TEM image in Fig. 2A illustrates the formation of long and straight nanoribbon like structures from TiO_2_ with a smooth surface which confirming the formation of TiO_2_ NRs during the hydrothermal treatment process. The length of the synthetic ribbons was ranged from 1 µm to a few micrometers, while the width was varied from ~20 nm to ~200 nm. During the growth of CNTs on the surface of TiO_2_(NRs) at a temperature of 700 °C, noticeable changes are observed in the surface morphology of the nanoribbons (Fig. 2B). This appeared in the formation of nano pits like structure with diameters ranging from 5 to 15 nm throughout the surface of TiO_2_ nanoribbons. Such nanopits give nanoporous like structure with the activated surface to the TiO_2_ NRs. The formation of the porous structure indicated the complete conversion of hydrogen titanate nanoribbons to TiO_2_-B nanoribbons and the presences of it as the single TiO_2_ phase in the synthesized composite^32^.

Figure 2C focus on the intergrowth of carbon nanotube with TiO_2_.B NRs in the synthetic nanocomposite. The intergrowth of CNTs exhibits different forms including their warping off around the ribbons, their growing within the surface of ribbons, and finally their separate growth without interfering with the nanoribbon. The formed carbon nanotubes are micrometer in length. The average outer and inner diameters of the tubes are 17 and 7 nm, respectively. The SEM image and EDX spectrum of TiO_2_ NRs/CNTs are shown in Fig. 2D. The composite composed of tube-like nanostructures forming an interlocked network from CNTs and TiO_2_-B NRs. The EDX spectrum indicated the presences of titanium, carbon, and oxygen signals as the components of the synthetic composite in addition to traces from the FeCO-Al_2_O_3_ catalyst. The quantitive EDX analysis revealed that the composite composed of 34.25% Ti, 34.02% C, 28.8% O, 1.73% Fe and 1.20% Co which confirm the synthesis of the composite at a mass ratio of about 1:1.

The specific surface area and the other textural properties of TiO_2_ NRs, CNTs, and TiO_2_ NRs/CNTs composites were estimated from the nitrogen adsorption/desorption isotherm curves, Fig. S1(supplementary data). The measured BET surface areas for TiO_2_ NRs, CNTs, and the TiO_2_ NRs/CNTs composite are 20.22, 86.48, and 102.75 m^2^/g, respectively. These data reflect the significant enhancement in the surface area of the synthesized composite compared to TiO^2^ NRs and CNTs. Also, the obtained pore volumes are 0.0227, 0.132, and 0.132 cm^3^/g indicating a considerable increase in the porosity of the composite as compared to pure TiO_2_ NRs. The average pore diameters for TiO_2_ NRs, CNTs and the composite are 5.74, 6.76, and 5 nm, respectively. This reveals the reducing the average pore diameter with the growth of CNTs over TiO2 NRs.

### FT-IR analysis

**Figure 3:**
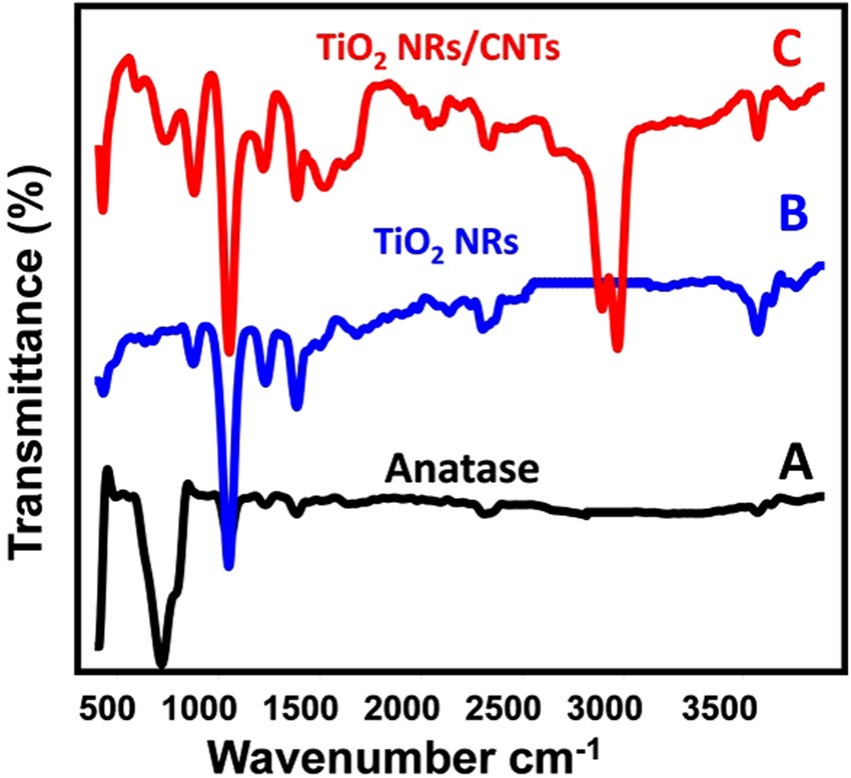
FT-IR spectra of the starting anatase TiO_2_ powder (**A**), Synthetic TiO_2_ NRs (**B**), and the synthetic TiO_2_ NRs/CNTs composite.

FT-IR technique was applied to detect the changes in the characterized functional groups with the transformation process (Fig. 3). The observed absorption bands and the related function groups of the starting TiO_2_ (Anatase) appear in Fig. 3A and Table 2. The broad absorption bands for the starting TiO_2_ powder around 710 cm^−1^ are assigned to Ti-O and Ti-O-Ti stretching vibration of anatase TiO_2_^27^. Other bands were observed at 1062, 1160, 1396, and 2332 cm^−1^ which are related to Ti-O, Ti-O-Ti, Ti-OH and O-H stretch from strongly hydrogen-bonded, respectively^19,33^.

**Table 2:** FT-IR analysis of the starting TiO_2_ powder, TiO_2_ nanoribbons, and TiO_2_ NRs/CNTs composite.

Anatase	TiO_2_ NRs	TiO_2_ NRs/CNTs	Assignment
-----------------	Above 3500 cm^−1^	Above 350 cm^−1^	Surface O-H groups and adsorbed water^36^
-----------------	----------------	2978.3 cm^−1^	aliphatic asymmetrical –CH_2_ stretching^37^
-----------------	----------------	2878.7 cm^−1^	Aliphatic symmetrical–CH2 stretching^38^
2332 cm^−1^	2324 cm^−1^	2352.3 cm^−1^	O-H stretch from strongly hydrogen-bonded^33^
-----------------	-----------------	1607.8 cm^−1^	C=C stretching bond, or a C=O bond^39^
1396.6 cm^−1^ 1062.54 cm^−1^ and 1160.6 cm^−1^	1396.71 cm^−1^ 1061.37 cm^−1^ and 1163.24 cm^−1^	1398.6 cm^−1^ 1063.8 and 1156.78 cm^−1^	OH bond of vibration of the surface adsorbed of Ti-OH^19^ Ti-O and Ti-O-Ti bonds^19,33^
----------------	886.83 cm^−1^	891.51 cm^−1^	Ti-O and Ti-O-Ti of TiO_2_-B^34^
710 cm^−1^ ---------------------------------	----------------- ----------------- 442.99 cm^−1^	---------------- 755 cm^−1^ 440.59 cm^−1^	Ti-O and Ti-O-Ti of anatase^27^ Ti-O-C functional group^26^ Ti-O and Ti-O-Ti of TiO2-B^34^

There are noticeable changes in the position and intensity of the characteristic absorption bands of anatase due to the hydrothermal transformation of it into TiO_2_-B and hydrogen titanate nanoribbons as shown in Fig. 3B and Table 2. The typical bands of TiO_2_-B were detected at 442 and 886 cm^−1^^34^. This is associated with a complete disappearance of the characteristic vibration mode of anatase at 710 cm^−1^ and increase the sharpness of the absorption bands at 1062 cm^−1^, 1160 cm^−1^, 1396 cm^−1^ and 2332 cm^−1^. Also, a clear peak was observed above 3500 cm^−1^ for the OH function group, which confirms the transformation of anatase to TiO_2_-B and hydrogen titanate. Moreover, this spectrum clearly reflects the role of hydrothermal treatment and acid washing in the introducing of H and OH group into the TiO_2_ structure.

The synthesis of TiO_2_ NRs/CNTs composite was confirmed by the existence of several carbon functional groups (C-H, C=C, and C=O) as shown in Fig. 3C and listed in Table 2. Additionally, the interaction between carbon nanotubes and TiO_2_ nanoribbons was confirmed by the appearance of Ti-O-C functional group at about 755 cm^−1^^26^. Such bond attributed to a covalent link between titanate and carbon nanotubes^35^. The characteristic bands of TiO_2_-B show high intensity in the composite compared to the synthetic nanoribbons which reflected the role of temperature in the complete transformation of hydrogen titanate to TiO_2_-B.

### Optical properties

**Figure 4:**
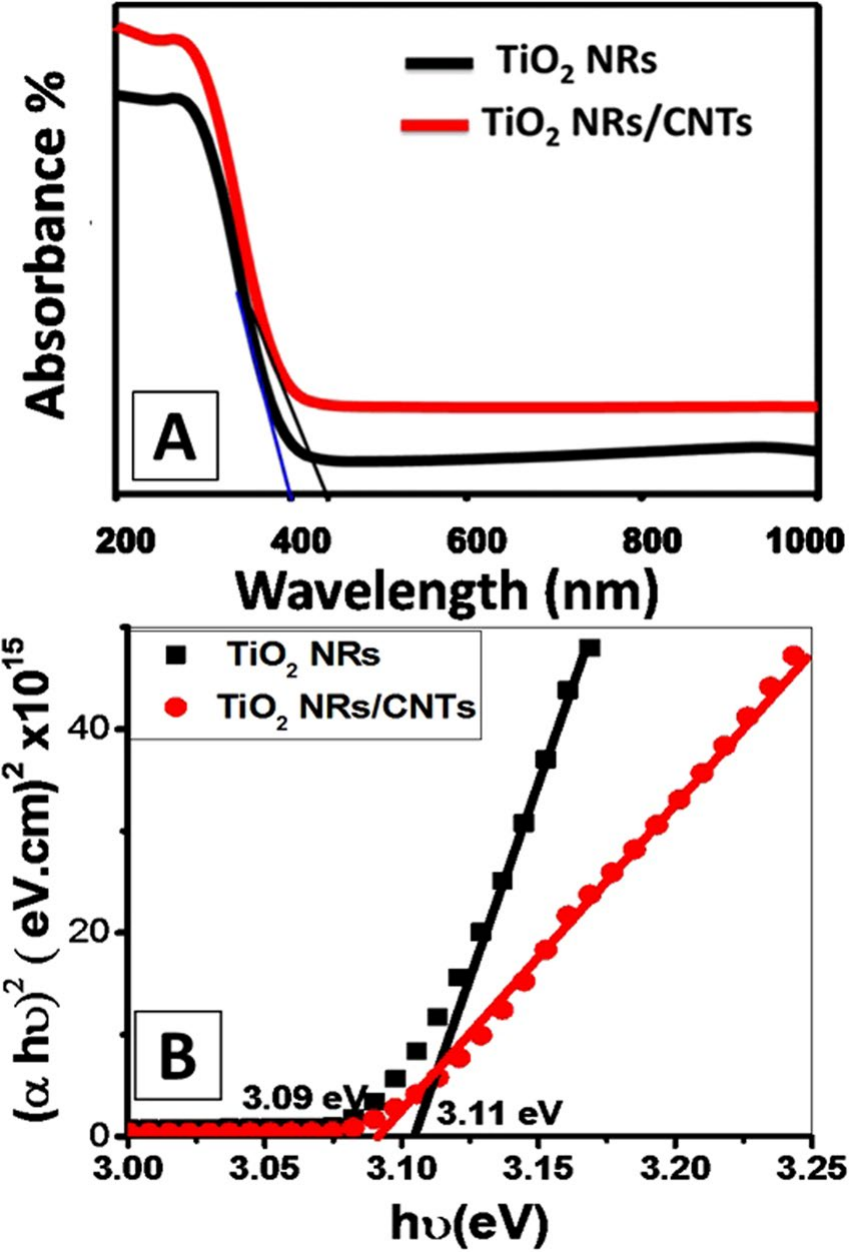
(**A**) UV-Vis absorbance spectra and (**B**) plot of (αh${\rm{v}}$)^2^ versus h ${\rm{v}}$ for band gap energy calculation for TiO_2_ NRs and TiO_2_ NRs/CNTs nanocomposite.

The study of optical properties of TiO_2_ NRs and TiO_2_ NRs/CNTs nanocomposite is one of the most important parameters that affect the applications of the synthetic products. The optical absorbance spectra of TiO_2_ NRs and TiO_2_ NRs/CNTs nanocomposite are shown in Fig. 4(A). Both samples displayed absorption bands within the UV region of the spectra. This was attributed to the intrinsic band gap absorption of titanium dioxide due to the electron excitation from the valence band (VB) to the conduction band (CB)^21^. However; there is noticeably enhancing in the absorbance of the TiO_2_ NRs/CNTs composite accompanied with red-shifting of the absorption edge as compared to that of TiO_2_ NRs. The detected shifting in the absorbance edge is related to the narrowing of the bandgap energy and reflected the chemical bonding between TiO_2_ NRs and the specific sites of carbon^40^.

Based on direct allowed transition type, the optical band gaps of all samples were estimated using Tauc’s equation (Eq. 2)^41^.

2 $$\alpha ={(hv-{E}_{g})}^{1/2}/hv$$

where 𝛼 is the absorption coefficient, A is the absorbance of the sample, *E*_*g*_ is the optical band gap, h is the Planck’s constant, *v* is photon frequency. The value of 𝛼 is given by Eq. 3^42^;

3 $$\alpha =2.303\times {10}^{3}A\beta /lC$$

where, β is the density of TiO_2_ NRs or TiO_2_ NRs/CNTs nanocomposite, *l* is the path of the quartz cell (1.0 cm), and C is the concentration of the powder in the suspension. The band gap value was determined by extrapolating the linear portion of (𝛼hν)^2^ versus h*v* to intercept with the h*v* axis as shown in Fig. 4(B). From this figure, the band gap values are 3.11 and 3.09 eV for TiO_2_ NRs and TiO_2_ NRs/CNTs nanocomposite, respectively. Reduction of band gap energy means speed excitation of electrons from the valance band to the conducting band utilizing low energy which will provide more electron/positive hole pairs and enhance the photocatalytic removal of the dyes^43,44^. However, the removal process of dyes and other organic pollutants is not controlled only by the value of band gap energy but also by the surface area and the density of adsorption active sites. This also reported for previously studied synthetic composites from graphene, graphene oxide and CNTs composited with TiO_2_ materials, which show small variations in the optical properties of the composite as compared to TiO_2_ precursor^12,15,17,18,37^. The small optical variations that noted for our composite may be related to the growth of CNTs within the pores of TiO_2_ NRs and over their surfaces which may reduce the interact between the incident light and surface of TiO_2_ as an active component for light. However, this cause great enhancement in the specific surface area and provide numerous active adsorption sites which is expected to improve the removal efficiency of dyes.

### Photocatalytic characterization

#### Effect of the irradiation time

**Figure 5:**
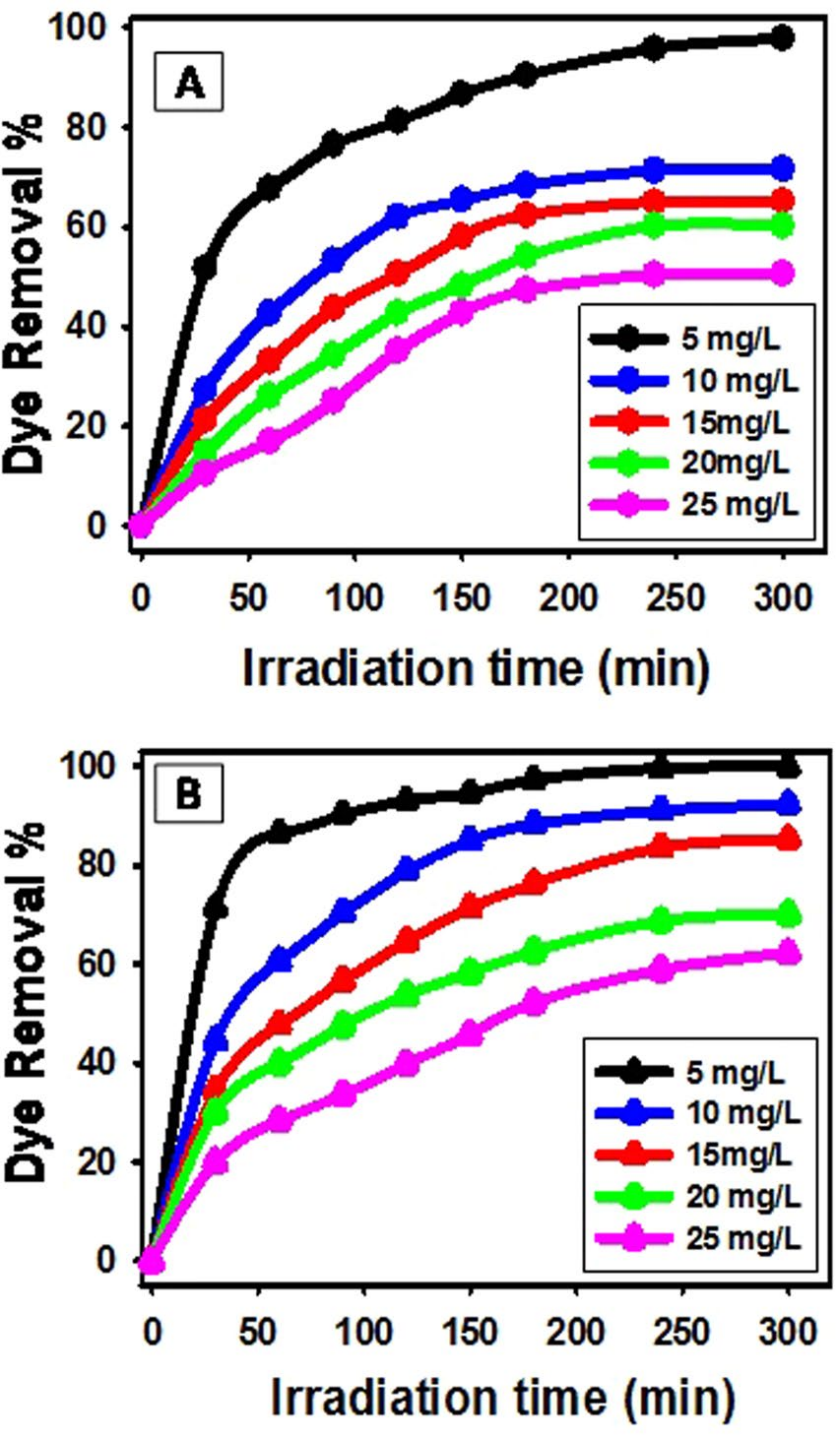
Effect of irradiation time on the degradation of different concentrations of methylene blue dye using (**A**) TiO_2_ NRs and (**B**) TiO_2_ NRs/CNTs nanocomposite.

The photocatalytic properties of TiO_2_ NRs and TiO_2_ NRs/CNTs nanocomposite were evaluated through their efficiency in degradation of methylene blue dye under sunlight. The photocatalytic degradation tests were performed at different dye concentrations within a time ranging from 30 to 300 min as illustrated graphically in Fig. 5.

The degradation of dye using only 0.02 g TiO_2_ NRs shows an increase in the degradation efficiency from about 51% to about 97.5% with increasing the irradiation time from 30 to 300 min (Fig. 5A) at an initial dye concentration of 5 mg/L. Figure S2 (supplementary data) shows the UV-Vis spectra of MB dye solution after sunlight irradiation for different time intervals using catalyst dose of 0.02 g. Also, the UV-Vis absorbance spectra for MB blank sample without any catalyst in dark and light are shown in Figure S2 (dashed lines). At the same irradiation time, the degradation efficiency decreases with increasing the initial dye concentration. For example, the degradation efficiency decreases from 67.8% to 16.92% using TiO_2_ NRs by increasing the initial dye concentration from 5 to 25 mg/l after an irradiation time of 60 min. Then, by increasing the initial dye concentration longer irradiation time intervals are required for the complete removal of the dye. Moreover, the degradation rate at the higher dye concentration appears to decrease with time especially after 180 min reflecting the consumption of hydroxyl radicals with time.

The degradation of dye using the synthetic TiO_2_ NRs/CNTs nanocomposite shows a noticeable enhancement in the photocatalytic decolorization of 5 mg/l methylene blue (Fig. 5B). As shown, the required irradiation time for 97.3% dye removal is reduced from ~300 min to ~180 min using TiO_2_ NRs and TiO_2_ NRs/CNTs nanocomposite, respectively. Approximately, the complete removal of 5 mg/l methylene blue required irradiation time >300 min using TiO_2_ NRs and >180 min using TiO_2_ NRs/CNTs composite. Such enhancement was detected at all the used initial dye concentrations. At 60 min, the degradation efficiency decreased from 86.5% to 28.4% using TiO_2_ NRs/CNTs composite by increasing the initial dye concentration from 5 mg/l to 25 mg/l. Where the degradation efficiency at 300 min exceed 92%, 85%, 70%, and 62% at initial dye concentrations 10, 15, 20, and 25 mg/L, respectively.

#### Kinetic reactions

First order and second order kinetic models were addressed to study the degradation behavior of methylene blue using TiO_2_ NRs and TiO_2_ NRs/CNTs composite. Equations of the studied kinetic models were expressed as in Eqs 4 and 5 for the first order, and second-order kinetic models, respectively^45^:

4 $$\frac{{dc}}{{dt}}=-{{k}}_{1}{c}$$

5 $$\frac{{dc}}{{dt}}=-{{k}}_{2}\,{{c}}^{2}\,$$

where C is the concentration of dye; k_1_ and k_2_ are the apparent kinetic rate constants of first- and second-order reaction kinetics, respectively; t is the reaction time. By integration Eqs 4 and 5, the linear form of the kinetic reactions for the first order and second order kinetic models can be given as^45^:

6 $${{C}}_{{t}}={{C}}_{0}{{e}}^{-{{k}}_{1}{t}}$$

7 $$\frac{1}{{{C}}_{{t}}}=\frac{1}{{{C}}_{0}}+{{k}}_{2}{t}$$

where, C_t_ is the concentration of MB dye after irradiation time (t).

**Figure 6:**
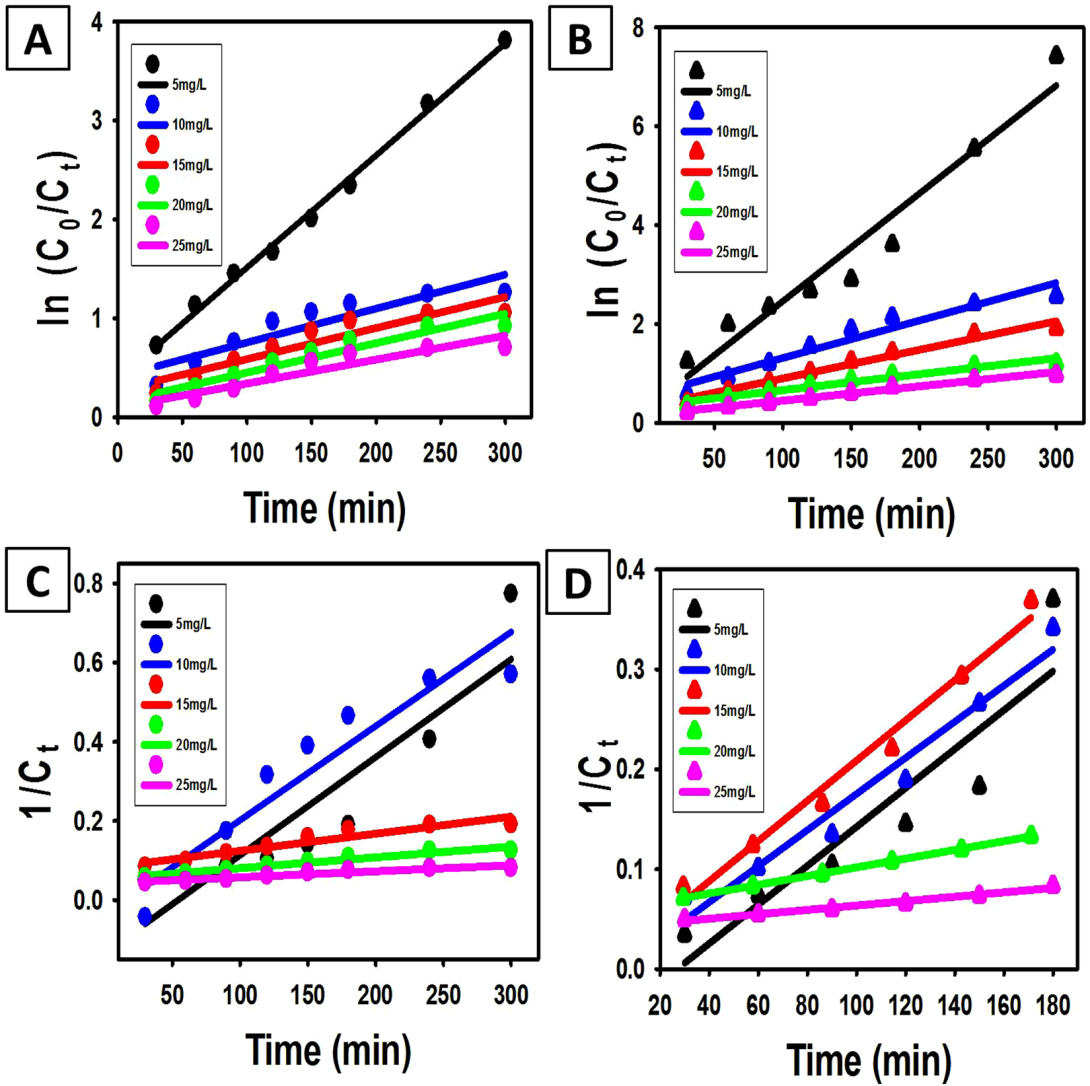
First order (**A,B**) and second order kinetic plotting (**C,D**) for the photodegradation of methylene blue using TiO_2_ NRs (**A,C**) and TiO_2_ NRs/CNTs nanocomposite (**B,D**).

The First order kinetic model was investigated from the linear relation between ln (C_0_/C) and t (Fig. 6A and B). The second order kinetic model was addressed from the linear relation between 1/C_t_ and t (Fig. 6C and D). The kinetic parameters for the selected models were listed in Table 3. The correlation coefficient (R^2^) is a measure that determines the degree to which two variables are related to each other. By comparing R^2^ for the two models, it can be concluded that the degradation of MB using TiO_2_ NRs is more fitted with the first order kinetic model than with the second-order kinetic model for low dye concentration (≤5 mg/L). But the degradation results at high MB concentrations (>10 mg/L) are more fitted with the second-order kinetic model than the first order kinetic model. This refers to a possible change in the operating degradation mechanism or the existence of more than one degradation mechanism at the high concentrations of MB.

**Table 3:** Parameters of first and second order kinetic models for dye degradation by TiO_2_ NRs and TiO_2_ NRs/CNTs nanocomposite.

Material	kinetic Model	Parameters	5 mg/L	10 mg/L	15 mg/L	20 mg/L	25 mg/L
TiO_2_ NRs	First order kinetic model	K_1_ (min^−1^) R^2^	0.011 0.996	0.0034 0.84	0.0031 0.87	0.003 0.86	0.0024 0.88
	Second order kinetic model	k_2_ (L/mol min) R^2^	0.029 0.83	0.0008 0.90	0.0004 0.91	0.0003 0.955	0.0002 0.908
TiO_2_ NRs/CNTs	First order kinetic model	K_1_ (min^−1^) R^2^	0.0281 0.95	0.0076 0.94	0.0058 0.976	0.0032 0.956	0.0029 0.938
	Second order kinetic model	k_2_ (L/mol min) R^2^	0.039 0.84	0.0045 0.962	0.0012 0.981	0.0004 0.999	0.0002 0.979

The values of kinetic degradation rate constants (K_1_ and K_2_) for the degradation of 5 mg/L dye concentration are higher than that calculated for the other initial MB concentrations (≥10 mg/L). Decreasing the degradation rate by raising the initial dye concentration may be related to the increased amount of adsorbed MB dye on the surface of the catalyst above the critical limit. Thus, available active sites on the catalyst surface will be reduced, and in turn, the photogenerated hydroxyl radicals will be decreased^46^. Additionally, increasing the initial dye concentration will act as a blocking surface between the incident photons and the catalyst surface. Hence, the degradation rate will decrease due to the decline in the number of absorbed photons by TiO_2_ NRs^47^.

The kinetic studies of MB degradations using TiO_2_ NRs/CNTs nanocomposite show results close to that were obtained for the degradation using TiO_2_ NRs. The degradation results for 5 mg/L MB are more fitted with the pseudo-first-order kinetic model than the second-order kinetic model. For MB concentration ≥10 mg/L, the photocatalytic degradation of dye using the composite is well fitted with both first order and second order kinetic models. However, it slightly more fitted with the second-order kinetic model than the first order kinetic model. Fitting of the degradation results with the two models refers to the simultaneous and parallel operating of the degradation mechanisms at MB concentrations ≥10 mg/L^48^. The calculated values of photocatalytic degradation kinetic constant rate for the degradation using TiO_2_ NRs/CNTs nanocomposite are higher than those for the degradation using TiO_2_ NRs. This implies the role of CNTs in the enhancement of the photocatalytic properties of the composite as compared to TiO_2_ NRs.

#### Effect of catalyst mass

**Figure 7:**
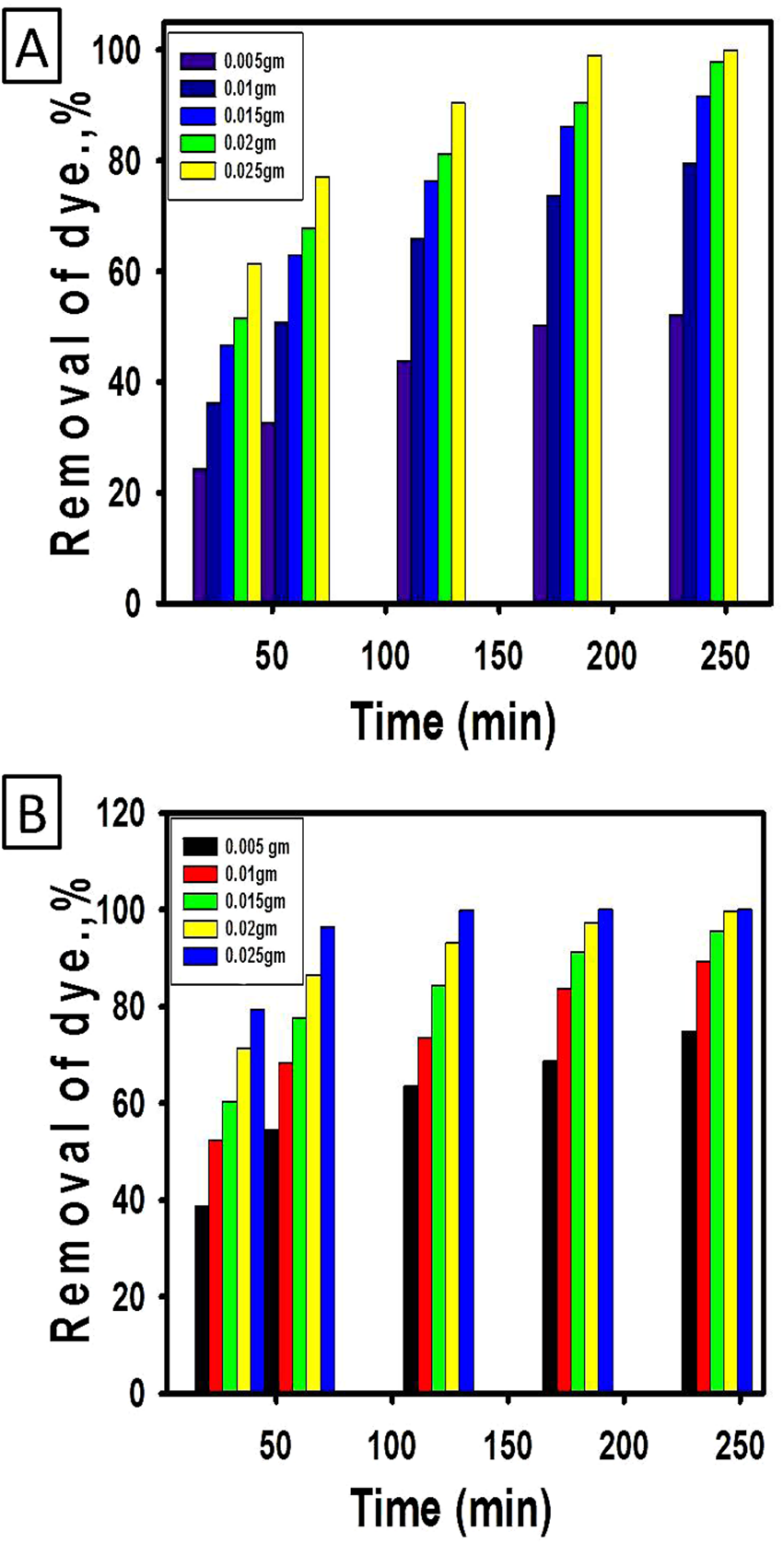
Effect of the catalyst dose on the degradation of methylene blue dye with time using TiO_2_ NRs (**A**) and TiO_2_/CNTs nanocomposite (**B**).

The effect of catalyst dose either TiO_2_ NRs or TiO_2_ NRs/CNTs composite on enhancing the photocatalytic removal percentage of methylene blue dye was evaluated at different time intervals and presented graphically in Fig. 7. For TiO_2_ NRs, the photocatalytic degradation of the studied dye exhibits an apparent increase in the removal percentage with increasing the applied dose from 0.005 to 0.025 g at all the studied time intervals (Fig. 7A). However, the effect of the applied dose on the removal percentage increases with increasing the irradiation time from 30 to 240 min. At the optimum irradiation time (240 min), the removal percentage increased from 52.13% to ~100% with increasing the applied dose from 0.005 g to 0.025 g, respectively. Increasing the removal percentage with increasing the applied dose might be credited to the related increase in the generated hydroxyl radicals and electron/hole pairs as well as the increasing of the adsorption capacity of the product with increasing surface area and the availability of more active adsorption sites^43,44,49^. The enhancement of the effect of the applied dose in the removal of dye with increasing the time intervals related to the increase in the number of excited electrons with increasing the irradiation time in addition to the associated increase in the amount of adsorbed methylene blue dye with increasing the contact time^43,44^.

The same behavior was detected for the photocatalytic removal of methylene blue dye using TiO_2_ NRs/CNTs composite. The removal percentage increased significantly with increasing the applied dose and the effect of the applied dose significantly enhanced with increasing the irradiation time intervals. For example, the removal percentage of the studied dye using 0.025 g of the composite increased to 79.3%, 96.47%, 99.94%, and 100% with increasing the time interval to 30, 60, 120, and 180 min, respectively. At all the studied catalysts dose and all the selected time intervals, the removal of the dye using TiO_2_ NRs/CNTs composite achieves higher efficiency as compared to the removal of the dye using TiO_2_ NRs.

#### Role of the catalyst support

**Figure 8:**
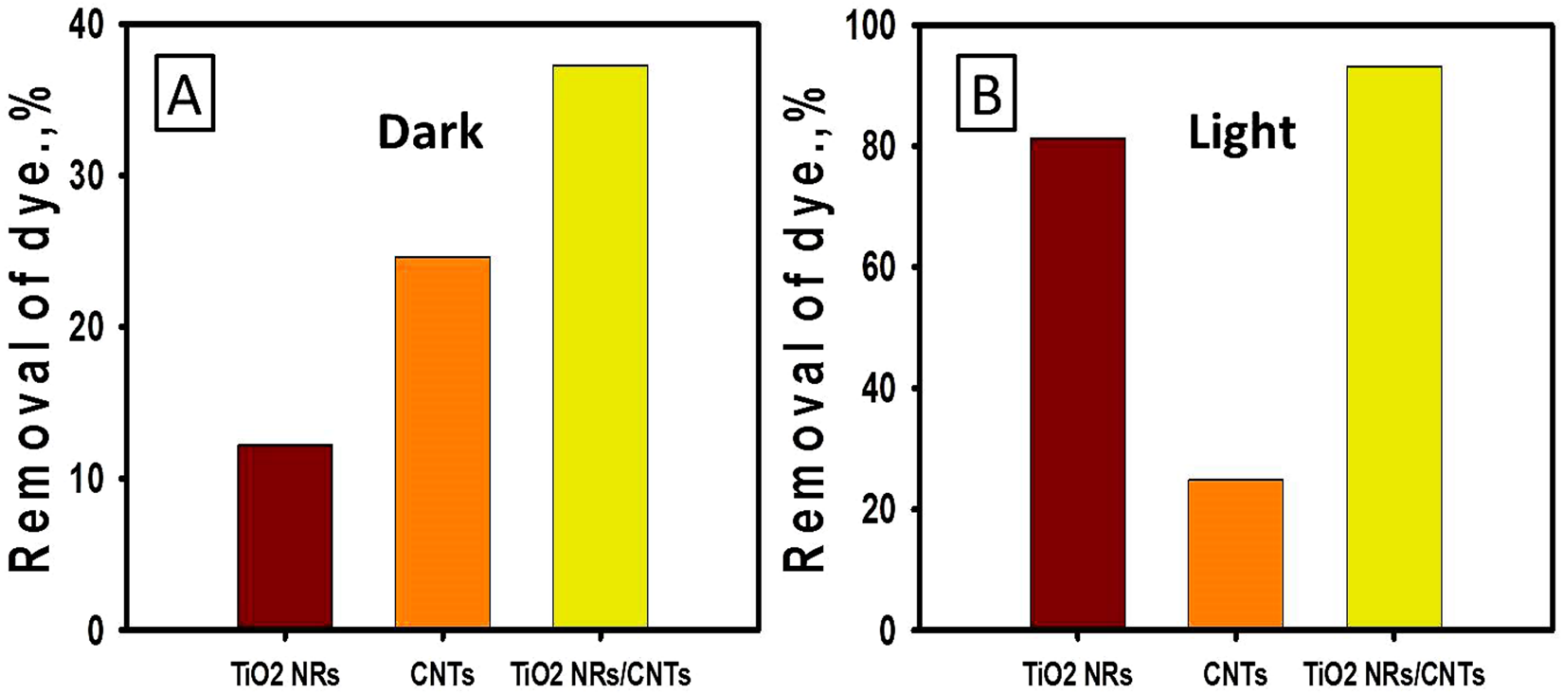
The removal of methylene blue dye through (**A**) adsorption (in the dark) and (**B**) photocatalytic degradation under sunlight irradiation using TiO_2_ NR, CNTs, and TiO_2_ NRs/CNTs nanocomposite.

It was reported that the presence of CNTs in hayride composite with semiconductor photocatalysts could enhance the adsorption capacity and increase the lifetime of the generated electron/hole pairs. To evaluate the role of CNTs in improving the adsorption capacity and photocatalytic properties of TiO_2_ NRs, the adsorption and photocatalytic removal of 5 mg/l methylene blue dye solution was evaluated after reaction time 240 min using the individual components as well as the composite. The adsorption removal% of methylene blue dye using TiO_2_ NRs, CNTs, and TiO_2_ NRs/CNTs composite was estimated to be 12.2%, 24.6%, and 37.3%, respectively (Fig. 8A). The obtained results indicated an evident enhancement in the adsorption capacity of the synthetic composite (TiO_2_ NRs/CNTs) as compared to the individual components (TiO_2_ NRs or CNTs) which reflected increases in the surface area and active adsorption sites after the growth of CNTs within the porous structure of TiO_2_ NRs.

Under the light irradiation, the produced CNTs as single phase show no noticeable increase in the removal percentage of methylene blue dye, while the removal of dye using TiO_2_ NRs and TiO_2_ NRs/CNTs composite show clear increase in the removal percentages to about 81% and 93%, respectively. I.e., there is an improvement in the removal% of methylene blue dye through photocatalytic degradation process by about 12% using the composite as compared to using TiO_2_ NRs as individual component (Fig. 8B). Hence the role of CNTs in enhancing the removal of methylene blue using the synthetic composite credited mainly to its role in increasing the adsorption capacity followed by its role in increasing the lifetime of the generated electron/hole pairs.

#### Degradation mechanism

**Figure 9:**
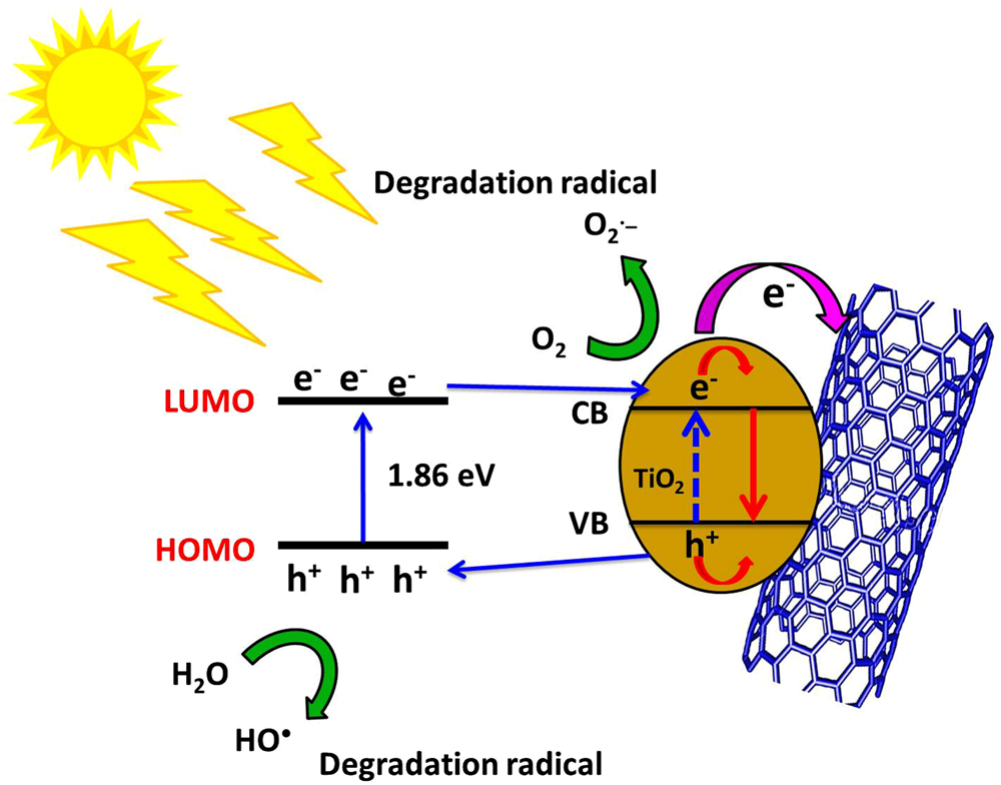
Schematic diagram for the photocatalytic degradation mechanism of MB dye using TiO_2_ NRs/CNTs nanocomposite.

The heterogeneous photocatalytic degradation involves three steps; (a) adsorption of the dye, (b) absorption of the light by the used catalyst, and (c) charge transfer reactions to generate the required radicals for dye degradation^14^. The degradation step can occur through direct degradation of dye by the photo created positive holes from the catalyst or through their role in the production of hydroxyl radicals^15^. The holes can oxidize the dye pollutants by direct electron transfer^50^. Also, the holes can react with an electron donor (water molecules or hydroxide ions) and produce oxidizing free radical (hydroxyl radical), which will oxidize dyes on the surface^51^. According to the degradation conditions, one of the previous mechanisms or both of them simultaneously contributes to the photocatalytic degradation process. Under the sunlight irradiation of TiO_2_ NRs, an electron is excited from the valence band to the conduction band to produce a positive hole that causes a decomposition of the present dye according to the previous mechanisms (Fig. 9).

The reported enhancement in the photocatalytic degradation of MB dye using the synthetic TiO_2_ NRs/CNTs nanocomposite is related to the interaction effect of CNTs. The presence of CNTs is facilitating the adsorption capacity of MB in the surface of the catalyst and increase the lifetime of the generated electron/hole pairs, or by both processes^15^. The later effect of CNTs is attributed to the energy level differences between TiO_2_ and CNTs. The conduction band position of TiO_2_ was determined to be at about 4.2 eV, and that of CNTs is about 4.7 eV depending on the number of graphene layers^52^. Thus CNTs act as an electron sink and the attached TiO_2_ NRs inject electrons from the conducting band to CNTs of the composite^14^. This resulted in a reduction of the probability of radiative electron−hole recombination. To control the electron/hole recombination properties, the photoluminescence (PL) spectra of TiO_2_ NRs, CNTs, and TiO_2_ NRs/CNTs are investigated to understand the behavior of the electron-hole pairs. Figure S3 shows the PL emission spectra of the TiO_2_ NRs, CNTs and TiO_2_ NRs/CNTs composite. It can be observed that the emission spectrum of TiO_2_ NRs/CNTs composite appears to be similar to that of CNTs. The emission peaks around 469 nm as shown in the inset of Fig. S3 are due to the free-excitation emission of the band gap and the charge transfer transition to the oxygen anion in a TiO_6_ octahedral complex from Ti^3+ ^^53,54^. Under the same excitation irradiation intensity, the emission intensity of TiO_2_ NRs/CNTs composite is lower than that of TiO_2_ NRs, suggesting that the recombination of photo-generated electrons/holes is efficiently inhibited. That is due to the work function of MW-CNTs (4.95 eV)^55^ is higher than that of TiO_2_ NRs (4.2 eV)^56^. Hence the photo-generated electrons will transfer from the conduction band of TiO_2_ NR to CNT until the Fermi level equilibrium is achieved, forming a Schottky barrier on their contact surface. This electron transfer can decrease the recombination probability of electrons and holes, leading to lower emission intensity^57^. Therefore, the use of TiO_2_ NRs/CNTs composite can reduce the recombination of electrons and holes and result in enhanced photocatalytic properties. I.e., lower value of the photoluminescence intensity corresponds to higher degree of photocatalytic degradation. This relationship was previously reported for the direct radiative and lower recombination of the generated carriers in TiO_2_^58^. However, the accurate explanation of photoluminescence - photocatalysis relationship needs more complex analysis of the different types of recombination, which occurs in CNTs and TNTs^58–60^. For the indirect radiative recombination, the photoluminescence intensity- photocatalytic activity relationship depends on the type of the incorporated dopant and oxygen vacancies at the surface, whereas the increase of defects and oxygen vacancies at surface produced higher photoluminescence intensity and photocatalytic activity^59^.

The generated electrons in CNTs can react with the present dissolved oxygen molecules producing oxygen peroxide (O_2_^**•**−^). Thus, MB dye pollutants can be degraded by the generated oxygen peroxide radicals or the effect of the formed holes in TiO_2_ NRs^19^. The photocatalytic degradation of MB dye using TiO_2_ NRs/CNTs nanocomposite and the predicted interaction between TiO_2_ and CNTs can be summarized in Eqs 8–15^19^:

8 $${Ti}{{O}}_{2}+{hv}\to {Ti}{{O}}_{2}(\,{{e}}_{{CB}}^{-},{h}_{VB}^{+}\,)$$

9 $${Ti}{{O}}_{2}({{\boldsymbol{e}}}^{-})+CNTs\to CNTs({e}^{-})+Ti{O}_{2}({e}_{CB}^{-},{h}_{VB}^{+})$$

10 $$Ti{O}_{2}({e}^{-})+{O}_{2}^{-}\to Ti{O}_{2}+{O}^{\bullet -}\,$$

11 $$CNTs({e}^{-})+{O}_{2}^{-}\to CNTs+{O}^{\bullet -}$$

12 $$Ti{O}_{2}({h}^{+})+{H}_{2}O\,\to Ti{O}_{2}+{H}^{+}+O{H}^{\bullet }$$

13 $$(Ti{O}_{2})e+MB\to (Ti{O}_{2}){e}^{-}+M{B}^{+\bullet }$$

14 $$M{B}^{+\bullet }+{O}_{2}^{-}\to Degradaed\,products$$

15 $$\,M{B}^{+\bullet }+O{H}^{\bullet }\to Degradaed\,products$$

The photocatalytic removal of methylene blue dye using TiO_2_ NRs/CNTs composite can be explained based on HOMO– LUMO (4.25 eV) gap value for methylene blue dye^61,62^. Under the effect of sunlight irradiation, a photogenerated electron can be excited from HOMO molecular orbital to LUMO molecular orbital in the prepared composite^62^. The excited photogenerated electron in the LUMO molecular orbital can be lost, while the HOMO molecular orbital requires critically one electron to return to the stable state. Hence, the required electron can be captured from water molecules, which cause oxygenation of water molecules into oxidation hydroxyl radicals. The generated hydroxyl radicals enhance efficiently the photocatalytic removal of methylene blue dye using the synthetic composite (Fig. 9).

**Figure 10:**
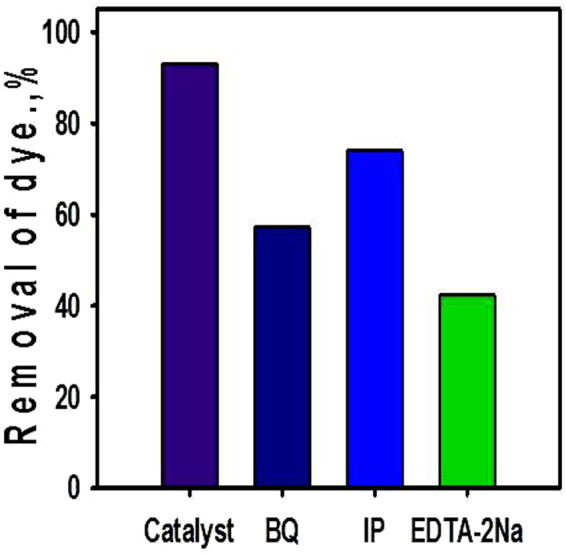
Trapping experiments of active oxidant species during the photocatalytic degradation of methylene blue dye over TiO2 NRs/CNTs composite.

For more explanation about the degradation mechanism of methylene blue dye using the synthetic composite, the active species trapping experiments were performed according to Chen *et al*.^63^. Based on the suggested experimental procedures, three chemical reagents (1 mmol of 1-4 benzoquinone (BQ), Isopropanol (IP), and Ethylenediaminetetraacetic acid sodium (EDTA-2Na)) were used as scavengers for the generated active radicals (superoxide, hydroxyl radicals and electron/hole pairs, respectively). The scavenging tests were carried out at fixed conditions of 5 mg/l dye concentration, 120 min irradiation time, and 0.02 g catalyst mass. The removal percentage of methylene blue dye decreased sharply from 93% to about 57.3% after incorporation of BQ in the photocatalytic system (Fig. 10). The addition of IP to the system resulted in a considerable decrease in the removal percentage from 93% to 74% (Fig. 10). Also, the addition of EDTA-2Na to the photodegradation system caused an apparent reduction in the removal percentage from 93% to 42.3% (Fig. 10). The obtained results indicated that the photocatalytic removal of methylene blue dye utilizing TiO_2_ NRs/CNTs composite controlled by the generated electron/hole pairs and the produced oxygen peroxide as the main active oxidants followed by the effect of the generated hydroxyl radicals.

#### Reusability

**Figure 11:**
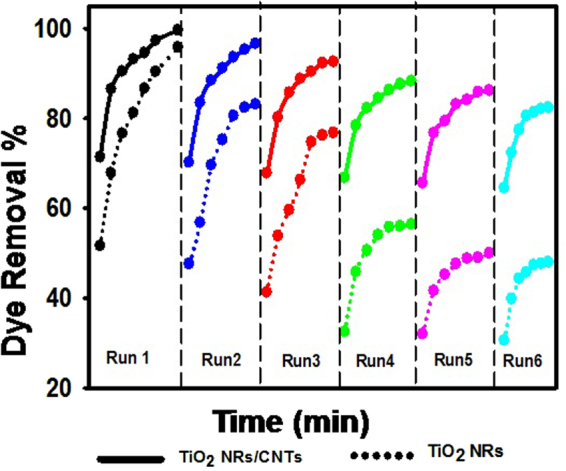
The reusability of TiO_2_ NRs and TiO_2_ NRs/CNTs nanocomposite for photodegradation of methylene blue for six runs.

The reusability (stability) is an essential factor for the photocatalyst practical application. The reusability of the catalyst for dye degradation was studied for six runs by stirring 0.02gm of catalysis with 5 mg/L methylene blue solution under sunlight within the time interval from 30 min to 240 min for each run. After each run, the catalyst powder was washed several times with distilled water and dried at 60 °C for 1 h before the next run. The degradation efficiency (dye removal %) using TiO_2_ NRs and TiO_2_ NRs/CNTs nanocomposite as a function of the number of runs is shown in Fig. 11.

Each run was carried out for 240 min. Although the degradation ratio of MB decreased slightly after each run, the TiO_2_ NRs and TiO_2_ NRs/CNTs catalysts exhibited ~ 48% and 81% degradation ratio at the sixth run, respectively. The degradation efficiency using TiO_2_ NRs is decreased from 96.1% at the first run to about 48% after the six run. So, the TiO_2_ NRs photocatalysts could remain 49.95% of the initial activity after six recycling runs. On the other hand, TiO_2_ NRs/CNTs photocatalysts showed higher stability than TiO_2_ NRs and could remain 82.6% of the initial activity after six recycling runs. This suggesting that the TiO_2_ NRs/CNTs has higher stability than TiO_2_ NRs photocatalyst and can repeatedly be used with very limited photo-corrosion.

## Conclusion

TiO_2_ NRs/CNTs nanocomposite was successfully synthesized by a chemical vapor deposition of CNTs using hydrothermally synthetic TiO_2_ NRs supported by the FeCo-Al_2_O_3_ catalyst. The synthesized TiO_2_ NRs and TiO_2_ NRs/CNTs nanocomposite were characterized by different techniques including XRD, FT-IR, TEM, SEM and UV-Vis spectrophotometer. The results reflected the transformation of hydrothermally synthetic TiO_2_-B and hydrogen titanate nanoribbon into a single phase TiO_2_-B NRs with nanopits structures. The single phase TiO_2_-B NRs was functionalized with FeCo-Al_2_O_3_ catalyst and composited with carbon nanotubes by the chemical vapor deposition method. The composite composed of tube-like nanostructures forming an interlocked network from CNTs and TiO_2_-B NRs. The composite exhibited stronger and broader optical absorption band than TiO_2_ NRs. Also, the band gap energy was reduced from 3.11 eV for TiO_2_ NRs to 3.09 for TiO_2_ NRs/CNTs nanocomposite. Moreover, the photocatalytic properties of TiO_2_ NRs and TiO_2_ NRs/CNTs composite were studied toward the photodegradation of methylene blue dye under sunlight. The effect of irradiation time, dye concentration, and catalyst dose on the photocatalytic properties of TiO_2_ NRs and TiO_2_ NRs/CNTs were addressed. The degradation mechanism and reaction kinetics were discussed. Using TiO_2_ NRs/CNTs composite, the irradiation time can be reduced to 50% compared to the same dose of TiO_2_ NRs. Moreover, TiO_2_ NRs/CNTs showed higher stability than TiO_2_ NRs photocatalyst and can repeatedly be used with very limited photo-corrosion.

## Materials and Methods

### Materials

TiO_2_ powder (CAS No. 13463-67-7), Fe (NO_3_)_3_·9H_2_O (CAS No. 7782-61-8), Co (NO_3_)_2_·6H_2_O (CAS No. 10026-22-9) and Al (NO_3_)_3_·9H_2_O (CAS No.7784-27-2) were purchased from Loba Chemie (India). HCl (36.6%) was purchased from scharlau(Spain). H_2_SO_4_ (98%) and HNO_3_ (69%) was used from SDFCL (India). Commercial C_2_H_4_ gas NH_4_OH, 32%, and NaOH flake were purchased from ADWIC (Egypt).

### Methods

TiO_2_ nanoribbons were synthesized by alkaline hydrothermal treatment for TiO_2_ powder. 4gm of TiO_2_ was mixed with 400 ml of NaOH solution (10 M) under stirring for 30 min. The resulted mixture then transferred into a Teflon-lined stainless autoclave and heated for 24 h at 170 °C. The solid was separated by centrifuge and washed with 0.1 M HCl and distilled water several times. The final product was dried at 80 °C for 4 h and partially calcinated at 450 °C for 2 h. Partial calcination was done to avoid the effect of further calcination during the other synthesis processes of the composite.

After partial calcination, the synthetic nanoribbons were loaded with a catalyst for CNT growth. FeCo-Al_2_O_3_ was used as a catalyst and synthesized according to Wen *et al*.^28^. Supporting of TiO_2_ nanoribbons by the catalyst was performed by coprecipitation of the catalyst on the ribbons surface. A certain amount of the synthetic TiO_2_ nanoribbons was dispersed in solution from iron nitrate, cobalt nitrate and aluminum nitrate under continuous stirring. Then, the solution PH was tuned to 8 using ammonia solution for complete precipitation of the catalyst. The catalyst-loaded TiO_2_ NRs were separated and washed several times with distilled water and dried at 80 °C for 4 h. Then the product was calcinated at 450 °C for 4 h to remove the excess of nitrate.

**Figure 12:**
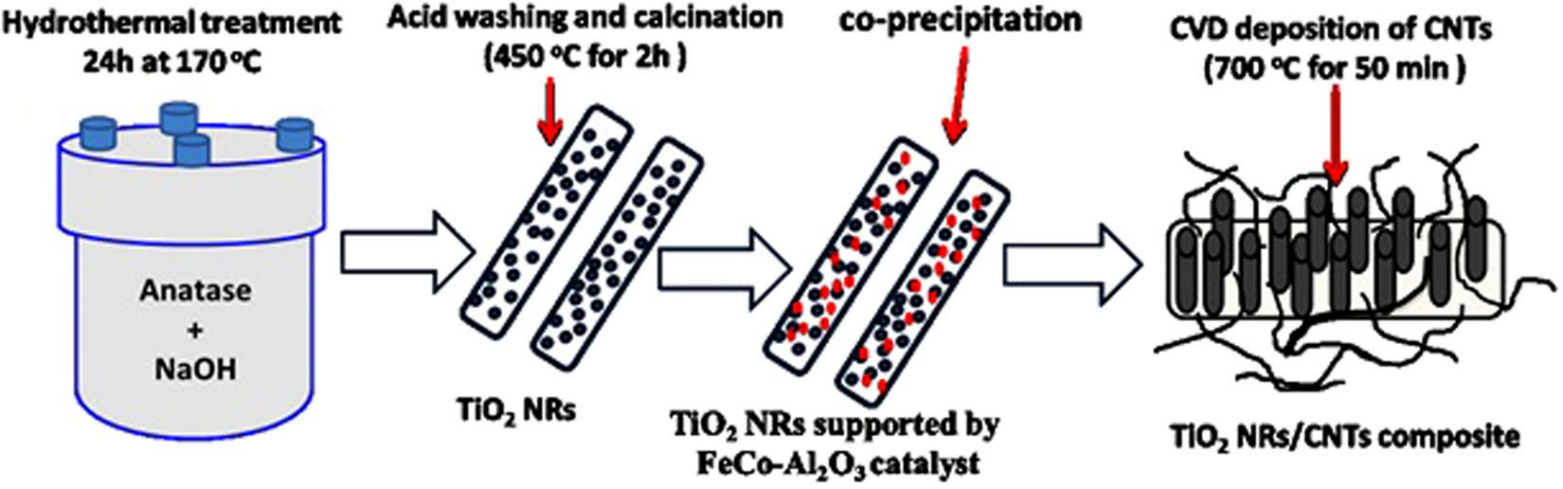
Schematic diagram of the synthetic steps of TiO_2_ NRs/CNTs nanocomposite.

Finally, TiO_2_ NRs/CNTs composite was synthesized using FeCo-Al_2_O_3_ –loaded nanoribbons as a catalyst for the growth of CNTs by chemical vapor deposition (CVD). A certain amount of this catalyst was set in ceramic and heated in a tubular electric furnace for 50 min at 700 °C under flux from C_2_H_4_ (carbon precursor) and nitrogen with ratio 1:10 v/v. The fabricated composite from CVD was added to H_2_SO_4_:HNO_3_ (1:3) in round bottom flask and refluxed for 6 h in oil path at 120°C to remove the catalysts. Then the obtained composite was washed with distilled water and dried at 80^o^C for 4 h as shown in Fig. 12.

### Characterization

X-ray powder diffraction patterns of TiO_2_ powder, TiO_2_ nanoribbons (TiO_2_ NRs), and the synthetic composite were obtained using a Philips APD-3720 diffractometer (Cu Kα radiation, operated at 20 mA and 40 kV) in the 2θ range of 5–70 at a scanning speed of 5°/min. Morphologies and structures of the synthetic materials were studied using a high-resolution transmission electron microscope (HR-TEM; Model 2010, JEOL, Tokyo, Japan) and scanning electron microscope (JSM-6510, JEOL, and Tokyo, Japan). The chemical composition was studied using energy dispersive X-ray (EDX; Oxford Link ISIS 300 EDX). The Fourier Transform Infrared spectrometer (FTIR − 8400 S Shimadzu, Japan) was used to determine the chemical structural groups of TiO_2_, TiO_2_ NRs, and TiO_2_ NRs/CNTs nanocomposite. The UV-visible absorption spectrum was measured using Shimadzu UV-vis spectrophotometer (M160 PC) at room temperature in the range 200–1000 nm using dimethyl sulfoxide (DMSO) as a solvent and reference. The specific surface areas, pore volumes, and pore widths of TiO_2_ NRs, CNTs, and TiO_2_ NRs/CNTs nanocomposite were determined using Brunauer–Emmett–Teller (BET) method.

### Photocatalytic degradation of methylene blue dye

Degradation of methylene blue (MB) dye was carried out to evaluate the photocatalytic efficiency of synthetic TiO_2_ NRs/CNTs nanocomposite. The degradation experiments were conducted under sunlight as a function of illumination time, the initial concentration of dye, and the catalysts dose. The stability of catalysts was addressed for several runs of degradation. All the photocatalytic removal percentages of the dye were calculated after subtracting the removal percentages obtained due to photolysis of the dye (1.6%) by the sunlight without the catalyst.

#### Effect of Time and Initial Dye Concentration

The effect of contact time and the MB dye concentration on the degradation efficiency was addressed by shaking 0.02 g of catalysts with 100 ml of the dye solutions of different concentrations (5-25 mg/L with increment 5 mg/L) for different time intervals from 0 to 300 min. All the experiments were performed under the natural sunlight. Then, the dye solutions were separated by centrifuge to determine the dye concentration by UV-vis spectrophotometer.

#### Effect of photocatalyst dose

Effect of the catalysts dose on the degradation of dye was tested by stirring different doses of it (0.005, 0.01, 0.015, 0.02 and 0.025 g) with 100 ml of the dye solutions (10 mg/L) as separated tests for a time interval from 0 to 300 min under the sunlight. Then, the dye solutions were separated by centrifuge to determine the rest dye concentration.

The degradation percentage of MB dye was calculated according to Eq. 16.

16 $${Degradation}\,({ \% })\,=\frac{{100}({{C}}_{{0}}\,\mbox{--}\,{{C}}_{e})}{{C}_{{0}}}$$

where, C_0_ and C_e_ are the dye concentrations in the initial solution and the solution after treating with the photocatalyst.

#### Role of the catalyst support

The role of CNTs in enhancing the adsorption and photocatalytic properties of TiO_2_ NRs/CNTs composite was evaluated through the mixing of 0.02 g of TiO_2_ NRs and TiO_2_ NRs/CNTs composite with 100 ml of methylene blue dye solutions (5 mg/l) as separated tests in the dark and under the sunlight for 120 min. The solid catalysts were separated by filtration, and the residual dye concentrations were estimated using UV-vis spectrophotometer.

#### Stability of the catalysis

The most critical factor for the practical utility of a catalyst is its reusability and stability during reactions. The reusability of the catalyst to degrade the dye for several runs was studied by stirring 0.02 g of catalysis with 5 mg/L MB solution. After each run, the catalyst powder was washed with distilled water and dried at 60 ^o^C for 1 h for re-using. This was repeated for six runs under sunlight within time intervals from 30 to 240 min.

## Electronic supplementary material

Supplementary data
