# Supplementary material for: TiO2 Nanoribbons/Carbon Nanotubes Composite with Enhanced Photocatalytic Activity; Fabrication, Characterization, and Application
Source: Sci Rep. 2018 Jan 15;8:781. doi: 10.1038/s41598-018-19172-w (PMC5768712; doi:10.1038/s41598-018-19172-w)
Supplement: Supplementary file 1 — Supplementary data [file 41598_2018_19172_MOESM1_ESM.pdf]

Supplementary Data

TiO_2 Nanoribbons/Carbon Nanotubes Composite with Enhanced Photocatalytic Activity; Fabrication, Characterization, and Application

Mohamed Shaban[1*], Abdallah M. Ashraf [1,2], Mostafa R. Abukhadra [1, 3]

[1] Nanophotonics and Applications Lab, Physics Department, Faculty of Science, Beni-Suef University, Beni-Suef 62514, Egypt

[2] Chemistry Department, Faculty of Science, Beni-Suef University, Beni-Suef 62514, Egypt.

[3] Geology Department, Faculty of Science, Beni-Suef University, Beni-Suef 62514, Egypt.

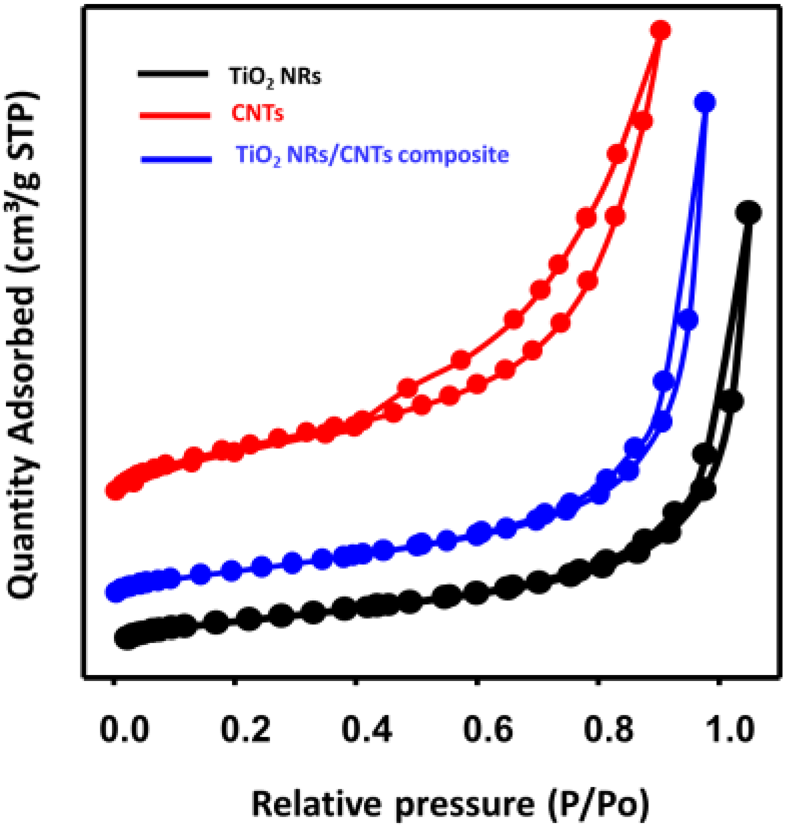

Fig. S1. The nitrogen adsorption /desorption isotherm curves of TiO_2 NRs, CNTs, and TiO_2 NRs/CNTs composites.

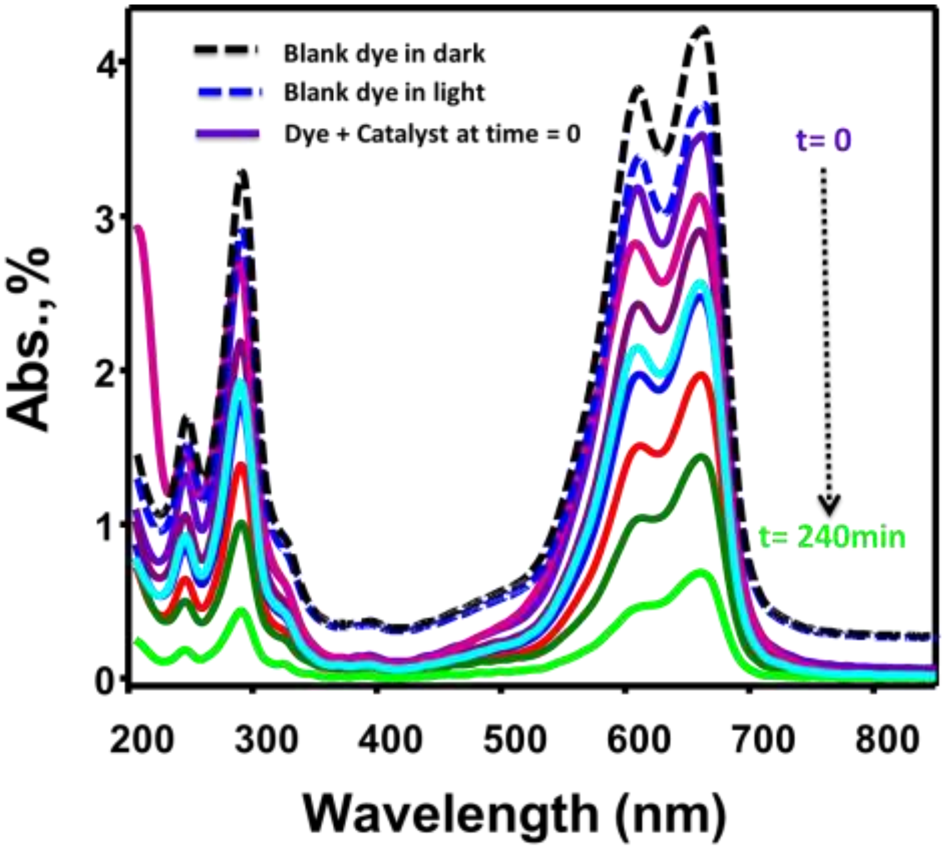

Fig. S2. UV-Vis spectra of MB dye solution after sunlight irradiation for different time intervals using a catalyst dose of 0.02g. The dashed lines spectra show UV-Vis absorbance spectra of MB blank sample without any catalyst in dark and light.

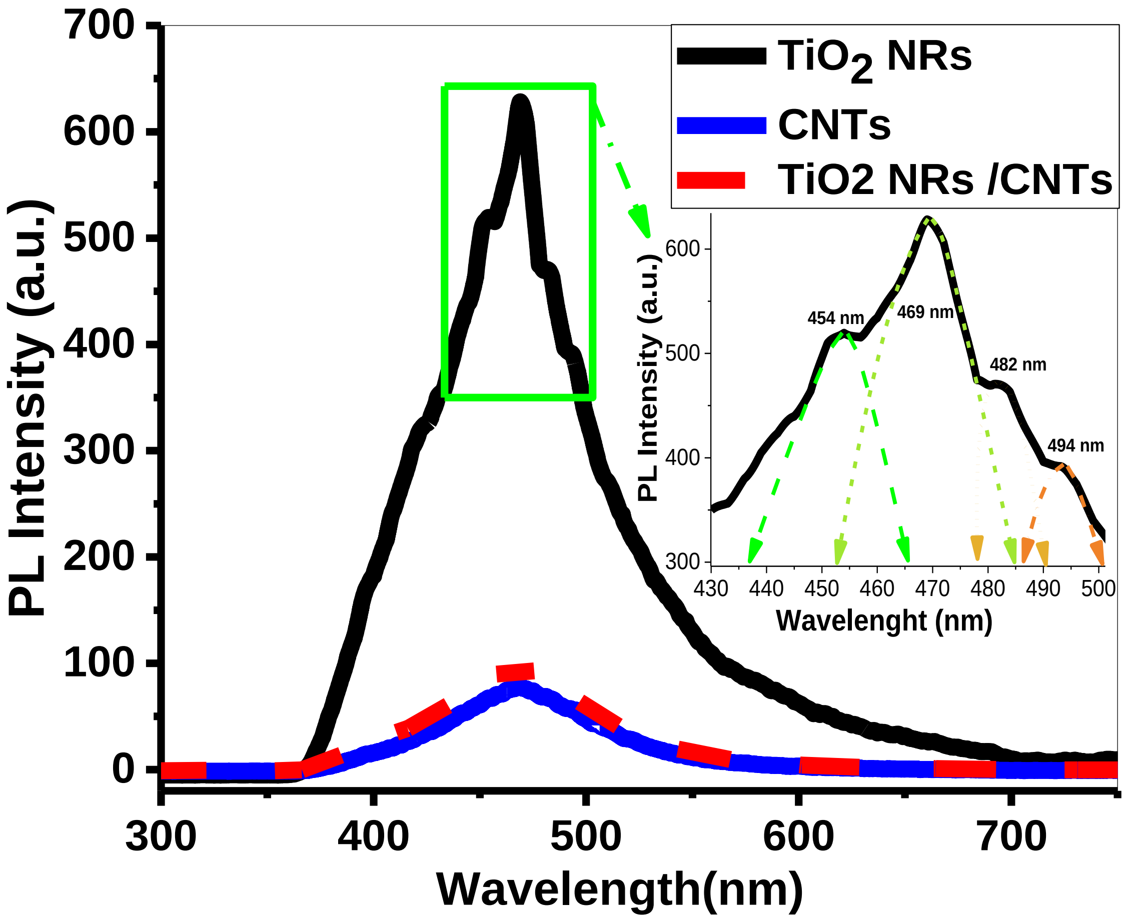

Fig. S3. PL spectra of TiO_2 NRs, CNTs, and TiO_2 NRs/CNTs.
